# Supplementary material for: Effect of Arbuscular Mycorrhiza Fungus Diversispora eburnea Inoculation on Lolium perenne and Amorpha fruticosa Growth, Cadmium Uptake, and Soil Cadmium Speciation in Cadmium-Contaminated Soil
Source: Int J Environ Res Public Health. 2023 Jan 1;20(1):795. doi: 10.3390/ijerph20010795 (PMC9819954; doi:10.3390/ijerph20010795)
Supplement: Supplementary file 1 [file ijerph-20-00795-s001.zip › ijerph-2048983-supplementary.pdf]

Table S1. Composition of full-strength Hoagland nutrient solution liquid

Properties	Values
$C_{10}H_{12}FeN_2NaO_8$ (mg/L)	20
K_2SO_4 (mg/L)	607
$MgSO_4$ (mg/L)	493
$(NH_4)_2SO_4$ (mg/L)	0.02
$ZnSO_4$ (mg/L)	0.22
$CuSO_4$ (mg/L)	0.05
$MnSO_4$ (mg/L)	2.13
$NH_4H_2PO_4$ (mg/L)	115
FeSO4 (mg/L)	2.86
$Na_2B_4O_7$ (mg/L)	4.5

Table S2. The effects of plant species (Plant), Cd, AMF and their interactions on shoot biomass, root biomass, total biomass, root shoot rate, pH, available phosphorus (A-P), neutral phosphatase, alkaline phosphatase, ammonium nitrogen (NH_4^+-N), nitrate nitrogen (NO_3^--N), shoot Cd concentration, root Cd concentration, shoot Cd accumulation, root Cd accumulation, total Cd accumulation, metal tolerance index, transfer factor, and bioconcentration factor by three-way ANOVA. Their P-values are indicated as ns (not significant), P > 0.05; *, P < 0.05; **, P < 0.01; ***, and P < 0.001.

Treatment	Plant	Cd	AMF	Plant × Cd	Plant × AMF	Cd × AMF	Plant × Cd × AMF
Shoot biomass	***	***	ns	ns	ns	ns	ns
Root biomass	***	ns	***	*	ns	*	**
Total biomass	***	***	**	*	ns	*	**
Root shoot rate	***	***	***	**	ns	***	***
pH	***	**	ns	*	ns	ns	ns
A-P	**	***	ns	***	ns	***	***
NH_4^+-N	***	ns	ns	ns	ns	ns	ns
NO_3^--N	***	***	***	***	***	ns	ns
Neutral phosphatase	***	ns	ns	ns	ns	ns	ns
Alkaline phosphatase	ns	ns	ns	ns	ns	**	ns
Exchangeable Cd	***	***	**	ns	ns	**	ns
Carbonate-Bound Cd	**	***	ns	ns	ns	ns	ns
Fe-Mn Oxides-Bound Cd	ns	*	ns	ns	ns	ns	ns
Organic-Bound Cd	ns	***	ns	ns	ns	ns	*
Residual Cd	**	***	***	ns	*	***	*
Shoot Cd concentration	***	***	ns	***	**	ns	ns
Root Cd concentration	***	***	ns	***	ns	ns	ns
Shoot Cd accumulation	***	***	ns	***	*	ns	ns
Root Cd accumulation	***	***	***	***	***	***	***
Metal tolerance index	***	***	***	ns	ns	**	***
Transfer factor	***	ns	*	ns	ns	ns	ns
Bioconcentration factor	***	*	**	ns	ns	ns	ns
